# Les altérations papillaires du myope fort

**DOI:** 10.11604/pamj.2014.17.130.3980

**Published:** 2014-02-24

**Authors:** Hakima Elouarradi, Rajae Daoudi

**Affiliations:** 1 Université Mohammed V Souissi, service d'ophtalmologie A de l'hôpital des Spécialités, Centre hospitalier universitaire, Rabat, Maroc

**Keywords:** Altérations papillaires, myopie, vice de réfraction, Papillary alterations, myopia, refractive error

## Image en médicine

En plus du vice de réfraction important occasionné, la myopie forte ou myopie axile maladie s'accompagne de modifications dégénératives des tissus oculaires notamment la sclère et la rétine responsables de la malvoyance et des différentes complications qui peuvent entraîner la perte fonctionnelle du globe oculaire, à savoir le glaucome, les lésions dégénératives de la périphérie rétinienne, la maculopathie myopique et le décollement de rétine. Le pôle postérieur du myope fort se caractérise par le staphylome, des modifications papillaires, une dégénérescence choriorétinienne évoluant vers l'atrophie, et des complications maculaires. Nous rapportons le cas d'une patiente myope présentant au fond d'oeil une atrophie épithéliale diffuse avec un conus myopique en temporal de la papille, correspondant à un glissement du complexe membrane de Bruch-épithélium pigmentaire-choriocapillaire . La papille est applatie avec absence de l'excavation physiologique. Tout patient myope nécessite une surveillance ophtalmologique régulière de son fond d'oeil avec pose de verre à trois miroirs.

**Figure 1:**
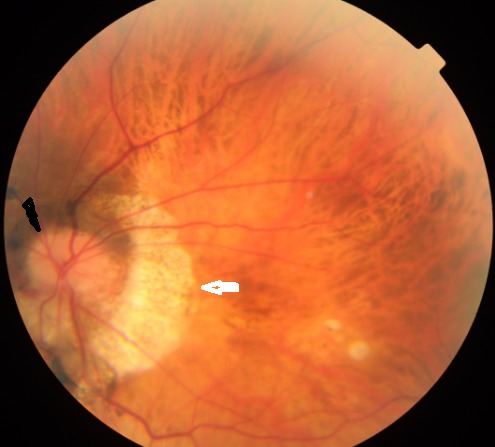
Fond d’œil du myope fort avec le conus myopique (flèche blanche) et une papille aplatie sans excavation physiologique (tête de la flèche noire)

